# Gemcitabine, Docetaxel, Capecitabine, Cisplatin, Irinotecan as First-line Treatment for Metastatic Pancreatic Cancer

**DOI:** 10.1158/2767-9764.CRC-23-0230

**Published:** 2023-08-28

**Authors:** H. Catherine Wilbur, Jennifer N. Durham, Su Jin Lim, Katrina Purtell, Katherine M. Bever, Daniel A. Laheru, Ana De Jesus-Acosta, Nilofer S. Azad, Bradley Wilt, Luis A. Diaz, Dung T. Le, Hao Wang

**Affiliations:** 1 Sidney Kimmel Cancer Center at Johns Hopkins University, Baltimore, Maryland.; 2 Memorial Sloan Kettering Cancer Center, New York, New York.

## Abstract

**Purpose:**

Treatment of advanced pancreatic cancer with a single therapeutic at a maximal dose has been largely ineffective at increasing survival. Combination therapies are commonly studied but often limited by toxicity. We previously showed that low-dose multiagent therapy with gemcitabine, docetaxel (taxotere), capecitabine (xeloda), and cisplatin (GTX-C) was safe, well tolerated, and effective (NCT01459614). Here, we hypothesize that adding irinotecan to GTX-C may improve survival with minimal toxicity.

**Experimental Design:**

Patients with treatment-naïve metastatic pancreatic adenocarcinoma were treated with gemcitabine, docetaxel (taxotere), capecitabine (xeloda), cisplatin, and irinotecan (GTX-CI). Treatment consisted of capecitabine 500 mg twice daily on days 1–14 and gemcitabine 300 to 500 mg/m^2^, docetaxel 20 mg/m^2^, cisplatin 15 to 20 mg/m^2^, and irinotecan 20 to 60 mg/m^2^ on days 4 and 11 of a 21-day cycle. The primary objective was 9-month overall survival (OS). Secondary objectives included response rate (RR), disease control rate (DCR), progression-free survival (PFS), and OS.

**Results:**

The regimen was well tolerated. The recommended phase II dose was gemcitabine 500 mg/m^2^, docetaxel 20 mg/m^2^, capecitabine 500 mg po bid, cisplatin 20 mg/m^2^, and irinotecan 20 mg/m^2^. Median follow-up in phase II was 11.02 months (2.37–45.17). Nine-month OS rate was 57% [95% confidence interval (CI): (41–77)]. RR was 57% [95% CI: (37–75) 50% PR and 7% CR]. DCR was 87% [95% CI: (69–96)]. Median OS and PFS were 11.02 [95% CI: (8.54–21.09)] and 8.34 [95% CI: (6.34–NA)] months, respectively.

**Conclusions:**

The addition of irinotecan to GTX-C was safe and well tolerated. While the study did not meet its primary objective, the responses were clinically meaningful using a well-tolerated regimen.

**Significance:**

We aimed to optimize the previously reported efficacious regimen of low-dose multiagent therapy with GTX-C for the treatment of metastatic pancreatic ductal adenocarcinoma by adding irinotecan. The primary objective was not met, but GTX-CI was well tolerated. The RR of 57%, median PFS of 8.3 months, median OS of 11 months, and 36-month OS rate of 19% suggest clinical benefit. Further optimization of this regimen is warranted.

## Introduction

Despite advances in systemic therapy for the treatment of pancreatic ductal adenocarcinoma (PDAC), 5-year overall survival (OS) remains 11% for all stages and a dismal 3% for patients with metastatic disease (1). The median OS and 1-year survival rate with gemcitabine and nab-paclitaxel, a commonly used first-line regimen for patients with metastatic PDAC, is 8.5 months and 35%, respectively (2). Because of treatment-related toxicities, the use of the alternative first-line regimen, FOLFIRINOX, is limited to patients with excellent functional status. The median OS and 1-year survival rate using FOLFIRINOX is 11.1 months and 48.4%, respectively (3). Recently reported, the median OS for NALIRIFOX, a multiagent regimen for the first-line treatment of metastatic PDAC, is 11.1 months (4). The treatment goal for patients with metastatic PDAC remains palliative with the intent of prolongation of life while minimizing toxicity. New therapeutic regimens must balance the potential for added toxicities while building on the knowledge that multiagent chemotherapy prolongs survival.

Several clinical trials have explored optimization of chemotherapy backbones. We previously presented results on a low-dose chemotherapy regimen combining gemcitabine, docetaxel, capecitabine, and cisplatin (GTX-C; ref. 5). Low-dose chemotherapy allows for the simultaneous emphasis on minimizing toxicity while utilizing multiple agents to battle tumor heterogeneity. Progression-free survival (PFS) at 6 months was 79% [95% confidence interval (CI): 0.65–0.95], exceeding the target primary endpoint of 75%. Furthermore, the objective response rate (RR; 50%), median OS (13.4 months), and 2-year OS rate (14%) were encouraging. Supporting the tolerability of the approach, there were no grade 3 or higher fatigue or neuropathy events. In comparison, the grade 3 or higher fatigue or neuropathy events occurred in 23.6% and 9% with FOLFIRINOX and 17% and 17% with gemcitabine/nab-paclitaxel (2, 3).

In the current study, we tested the hypothesis that a low-dose, five-drug, chemotherapy combination was safe and efficacious in the treatment of metastatic PDAC. To harness the benefit of a topoisomerase inhibitor with a different mechanism of action than the agents already in the GTX-C regimen, irinotecan was added to the chemotherapy strategy described above to prolong the time to therapeutic resistance.

## Materials and Methods

### Study Participants

The study was a single-center study performed at the Sidney Kimmel Cancer Center at Johns Hopkins University (JHU; Baltimore, MD). The protocol and all amendments were approved by the JHU Institutional Review Board and was conducted according to the Declaration of Helsinki and the guidelines for Good Clinical Practice. All patients provided written informed consent prior to participation in the study.

Eligible patients were 18 years or older, had histologically or cytologically confirmed untreated metastatic PDAC, measurable disease using RECIST v.1.1, Eastern Cooperative Oncology Group (ECOG) ≤ 1, and adequate organ and marrow function. Exclusion criteria included chemotherapy within 5 years, prior radiotherapy, age ≥ 76, major surgery within 28 days, or a known history of human immunodeficiency virus, hepatitis B, or hepatitis C.

### Study Design and Treatment

This open-label phase I/II study evaluated the efficacy and safety of gemcitabine, docetaxel, capecitabine, cisplatin, and irinotecan. All were administered intravenously on days 4 and 11 every 21 days except for the capecitabine, which was administered at a dose of 500 mg orally twice daily days 1 through 14 every 21 days. Dose levels 1, 2, 3 had fixed doses of gemcitabine 400 mg/m^2^, docetaxel 20 mg/m^2^, cisplatin 15 mg/m^2^, and escalating doses of irinotecan 20, 40, and 60 mg/m^2^, respectively (Supplementary Fig. S1). The study was then amended to add levels 1a and 1b, which had fixed doses of gemcitabine 500 mg/m^2^, docetaxel 20 mg/m^2^, cisplatin 20 mg/m^2^, and escalating doses of irinotecan 20 and 40 mg/m^2^. Dose level 1a was chosen for expansion.

Patients received intravenous premedication with dexamethasone 12 mg, ondansetron 8 mg, and fosaprepitant 150 mg i.v., and 500cc intravenous normal saline before and after cisplatin infusion. Oral dexamethasone and ondansetron were administered the second and third day after each intravenous infusion. Filgrastim was not prophylactically administered but was allowed if deemed necessary by the investigator.

### Assessments

Physical examinations were performed every cycle. Adverse events (AE) were graded according to the Common Terminology Criteria for Adverse Events, v.4.0. Hematology and chemistry laboratories were recorded as AEs if ≥ grade 3 or deemed clinically significant. CT or MRI were performed at baseline and every 9 weeks. RECIST measurements were performed by radiologists. Tumor markers, carbohydrate antigen 19-9 (CA19-9) and CEA, were performed at baseline and the beginning of every cycle. Patients could continue treatment despite progression if the principal investigator felt that the patient was clinically benefiting but were taken off study if progression continued.

### Statistical Analysis

The phase I portion evaluated five dose levels (Supplementary Fig. S1) in a 3+3 design to determine the recommended phase II dose (RP2D). The primary objective of phase II of the study was OS at 9 months defined as the proportion of patients alive at 9 months. The treatment regimen would be considered of insufficient activity for further study if the 9-month OS rate was 57% or less. The minimum required level of efficacy that would warrant further study with the proposed regimen was an 80% OS rate at 9 months. A two-stage design based on the Green and Dahlberg method (6) was used. A total of 30 patients treated at RP2D (15 patients in the first stage) had 90% power with one-sided type I error of 0.1. If a total of 22 or more patients were alive at 9 months, then the regimen would be considered promising. Safety analysis included all patients who received any treatment. Efficacy analysis included all patients who received any treatment at RP2D. OS and PFS were summarized using Kaplan–Meier method. RR was estimated as the proportion of patients who achieved complete response (CR) or partial response (PR) per RECIST 1.1 in phase II. Disease control rate (DCR) was the proportion of patients who achieved CR, PR, or stable disease (SD) at 8 weeks. Exact binomial 95% CI was calculated for proportions.

### Data Availability

The data generated in this study are available within the article and its Supplementary Data.

## Results

### Patient Characteristics

Forty-eight patients were enrolled between February 2015 and September 2017. Representativeness of study participants is provided in Supplementary Table S1. One patient did not receive treatment because of clinical deterioration. Dose-level assignment is listed in the CONSORT diagram (Supplementary Fig. S1). Baseline characteristics of the expansion cohort are listed in Table 1. The median age was 65 years old. A majority of patients (87.2%) had liver metastases. Twenty-three percent of patients had ascites.

**TABLE 1:** Baseline characteristics

	Phase I dose escalation *N* = 17	Phase II dose expansion *N* = 30	Total *N* = 47
Age, years			
Median (range)	63 (37–73)	65 (42–74)	64 (37–74)
≥65	7	15	22
Sex			
Female	9	11	20
Male	8	19	27
Race			
Asian	1	1	2
Black	2	3	5
White	14	26	40
Ashkenazi Jewish descent	1	4	5
ECOG			
0	4	11	15
1	13	19	32
Baseline CA19-9			
Normal	3	5	8
Elevated	14	25	39
Primary tumor location on pancreas			
Head/Neck/Uncinate	8	14	22
Body/Tail	9	16	25
Differentiation			
Moderately	4	13	17
Poorly	12	14	26
Unknown	1	3	4
Site of metastases			
Liver	16	25	41
Lung only	0	0	0
Baseline ascites			
Yes	4	7	11
No	13	23	36
NLR			
≤5	13	23	36
>5	4	7	11

Abbreviation: NLR, neutrophil lymphocyte ratio.

### Treatment Exposure and Dose Selection

While there were no dose-limiting toxicities (DLT) in level 1, there was 1 patient that experienced two DLTs of grade 4 renal failure and grade 3 hypophosphatemia in dose level 2 (the fourth patient in the cohort), and enrollment in that cohort was stopped. Two of 4 patients in dose level 3 had grade 3 diarrhea. However, the grade 3 event lasted less than 72 hours and did not meet DLT criteria. One DLT of grade 3 neutropenia lasting >7 days occurred at dose level 3. The protocol was amended to add levels 1a and 1b to increase gemcitabine but decrease irinotecan dosages. While there were no DLTs in level 1b, 2 of 3 patients had grade 2 or 3 diarrhea. Therefore, dose level 1a was chosen for dose expansion. Thirty patients were included in the expansion cohort (6 from the dose escalation + 24 additional patients). The median number of cycles of therapy was 12 (range: 1–33). Twenty-two patients (73%) received treatment for at least 6 months, 11 patients (37%) for at least 12 months, 4 patients (13%) for at least 18 months, and 4 patients (13%) for at least 24 months. Fifteen patients (50%) required at least one dose reduction; however, a median of 8 cycles were received before a dose reduction was required. Cisplatin was discontinued in 8 patients after 1, 6, 8, 9, 11, 16, and 18 cycles, respectively, due to rise in creatinine. Irinotecan was discontinued in 5 patients after 3, 4, 5, 11, and 15 cycles, respectively, due to bone marrow suppression or diarrhea. Capecitabine was dose reduced in 4 patients for hand-and-foot syndrome and thrombocytopenia. Gemcitabine was dose reduced for 1 patient due to grade 3 vomiting.

### Safety and AEs

Overall, the gemcitabine, docetaxel (taxotere), capecitabine (xeloda), cisplatin, and irinotecan (GTX-CI) dose selected for the expansion phase portion of the trial was well tolerated. The most frequent grade 3 or higher treatment-related AEs were anemia (60%), neutropenia (60%), and leukopenia (47%; Supplementary Table S2). Creatinine increases were seen in 8 patients (grade 1–2). Neutropenic fever occurred in 2 patients. There were no incidences of grade 3 or higher fatigue or neuropathy. Twenty patients (67%) required magnesium supplementation. Grade 4 events included hyperkalemia (3%), hypokalemia (3%), elevated lipase (3%), leukopenia (7%), lymphopenia (10%), neutropenia (37%), and thrombocytopenia (13%). There were no treatment-related deaths.

### Efficacy

#### Survival and Response

**FIGURE 1:**
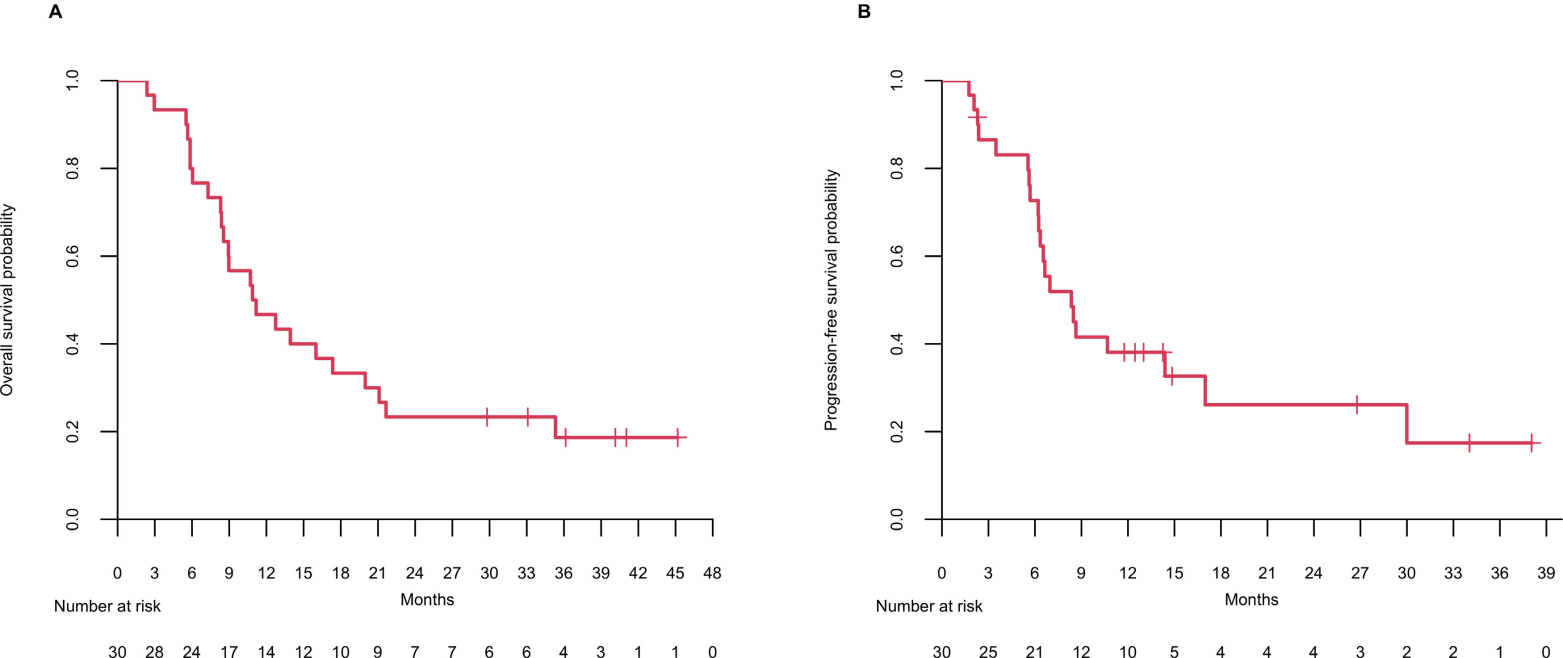
Kaplan–Meier estimates of OS and PFS in the expansion cohort. **A,** shows OS; the median was 11 months. **B,** shows PFS; the median was 8.3 months.

The data cutoff for analyses was March 2, 2020. The study enrolled a total of 30 patients at RP2D. The median follow-up for the expansion cohort was 11.02 months [range: (2.37–45.17)]. The Kaplan–Meier OS at 9 months was 57% (17/30 patients, 95% CI: 41–77; Fig. 1A). The study did not meet the proposed minimum required level of efficacy of an 80% OS rate at 9 months to warrant further study. The median OS time was 11 months (95% CI: 8.5–21.1). The 6-, 9-, 12-, 18-, 24-, 30-, and 36-month OS rates were 80%, 57%, 47%, 33%, 23%, 23%, and 19%, respectively. The median PFS was 8.3 months (95% CI: 6.3–NA; Fig. 1B). The RR was 56.7% (95% CI: 37.4–74.5; 17/30). There were 2 CRs and 15 PRs. The DCR was 86.7% (95% CI: 69.3–96.2; 26/30; 2 CR + 15 PR + 9 SD; Table 2). Six patients (20%) underwent resection to render disease status to no evidence of disease. Two of these patients had a pathologic CR. Survival time and patient characteristics are provided in Supplementary Table S3. Of the 5 patients who were alive at the March 2020 data cutoff, 3 have remained off chemotherapy for 23, 27, and 29 months.

**TABLE 2:** Objective responses in the expansion cohort

	Overall (*N* = 30)
Objective response	
CR	2.00 (6.7%)
PR	15.0 (50.0%)
SD	9.00 (30.0%)
PD	3.00 (10.0%)
NE	1.00 (3.3%)

Abbreviations: PD, progressive disease; NE, non-evaluable.

Results of univariate logistic regression analysis did not reveal any variables to be associated with response; however, univariate and multivariate analyses identified the log baseline neutrophil count to be associated with poor OS [HR, 4.2 (95% CI: 1.0–17.7); *P* = 0.047]. There was no evidence of statistically significant association between differentiation status and survival outcomes. The median OS, median PFS, and RR based on differentiation status were 13.9 months, 8.5 months, and 77% (DCR 92%), respectively, for those with well to moderately differentiated tumors and 10.8 months, 8.6 months, and 43% (DCR 86%), respectively, for those with poorly differentiated tumors (Supplementary Fig. S2). The 24-month OS rate was approximately 20% in both groups (23% for those with well to moderately differentiated tumors and 29% for those with poorly differentiated tumors).

#### Tumor Markers

**FIGURE 2:**
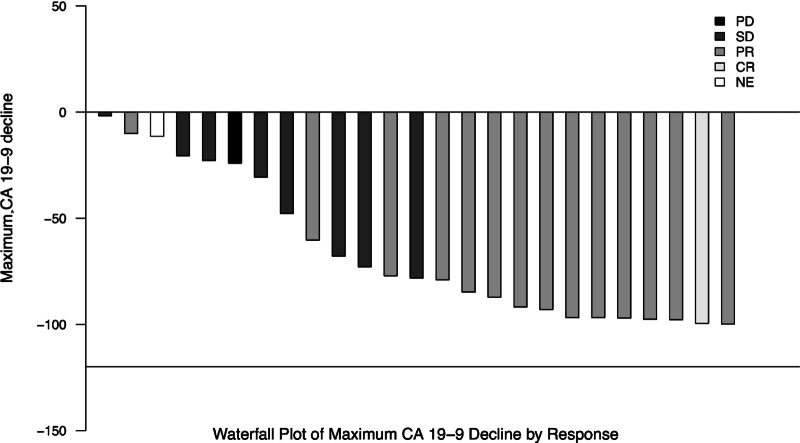
Maximum CA 19-9 responses in the expansion cohort. Figure shows the CA 19-9 percent change from baseline according to response of progressive disease (PD), SD, PR, CR, or not evaluable (NE).

The tumor markers, CEA and CA19-9, were collected at baseline and with the start of each treatment cycle. In the expansion cohort, baseline CEA was elevated above the upper limit of normal in 100% of patients with a median value of 8.4 ng/mL (range: 1.6–2,051). Baseline CA19-9 was elevated above the upper limit of normal in 83% (25/30) of patients with a median value of 630 U/mL (range: 42–152,256). The maximum CA19-9 decline for those 25 patients is shown in Fig. 2.

## Discussion

This study tested the safety and efficacy of gemcitabine, GTX-CI in treatment-naïve metastatic PDAC, a patient population in which combination chemotherapy is the cornerstone of management but often limited by cumulative toxicity, poor tolerability, and chemotherapy resistance. Despite not reaching the ambitious goal of an OS rate of 80% at 9 months, the RR of 57%, median PFS of 8.3 months, median OS of 11 months, and 36-month OS rate of 19%, without any reports of grade 3 or greater fatigue or neuropathy, suggests the value of this approach. Our regimen limits the toxicity of treatment and increases tolerability, providing a treatment option that warrants additional optimization.

Nine-month OS as the primary outcome was intentionally chosen to assess the benefit of GTX-CI for the majority of patients. Historically, 9-, 24-, and 36-month OS rates with gemcitabine and nab-paclitaxel were approximately 48%, 9%, and <5% and with FOLFIRINOX were 57%, 9%, and <5% (2, 3). In the current study, the 9-, 24-, and 36-month OS rates were 57%, 23%, and 19%. In fact, the OS rate nearing 4 years remains 19%. This plateau in the curve suggests that a subset of patients have a remarkably durable response to this therapy. Given the sensitivity of PDAC with homologous recombination deficiency (HRD) to cisplatin, we could postulate that this molecular underpinning could account for these survivors (7). Of the 16 patients tested for germline mutations in *BRCA1* and *BRCA2*, 2 patients (12.5%) were found to carry a pathogenic germline mutation in either *BRCA1* or *BRCA2;* both patients experienced a PR to treatment (Supplementary Table S3). Despite this low prevalence in our patient population, there is evidence to suggest that not all patients with PDAC with a DNA damage repair signature can be identified by clinical sequencing and may instead be selected based upon clinical phenotype, such as sustained platinum sensitivity (8). We again could postulate that this subset of patients composes the patients with prolonged survival. Alternatively, this regimen has efficacy despite HRD. In addition, 13 patients (27.7%) were found to have a KRAS mutation, all of whom experienced either a PR (*n* = 7, 53.8%) or SD (*n* = 6, 46.2%). Further investigation is needed to identify a molecular subset of patients who may respond favorably to this regimen.

Another interesting facet of this study is that 6 patients underwent surgical resection after a range of 11 to 27 months of chemotherapy. Of those, 3 patients (50%) had biopsy proven metastases at time of diagnosis (2 liver, 1 osseous). The remaining 3 had biopsies of the pancreas consistent with pancreatic adenocarcinoma and imaging findings to support the diagnosis of metastatic PDAC. One had 2[18F]fluoro-2-deoxy-D-glucose–avid liver metastases at diagnosis. One had a liver metastasis on CT and 1 had a peritoneal implant on CT; both sites of metastases responded to treatment. However, in the 10 patients with the longest survival (range: 20+ to 45+ months), 4 patients did not undergo surgery. Surgery was not part of the study recommendations, and the decision was made at the discretion of the primary team. This again highlights a subset of patients with a durable and robust treatment response who warrant further characterization.

While this study was small, the median OS of 11 months, though better than the 8.5 months expected with gemcitabine/nab-paclitaxel, was not numerically superior to the 11.1 months with FOLFIRINOX or NALIRIFOX or the 13.4 months with GTX-C (NCT01459614; refs. 2–5). However, the 2-year and 4-year OS rates of approximately 19% for GTX-CI are provocative. There is the possibility that irinotecan may prolong survival for patients with poorly differentiated tumors. With the GTX-CI regimen, 2-year survival was similar regardless of differentiation status. In patients with documented differentiation status, poorly differentiated tumors accounted for 52% (14/27) of cancers in the GTX-CI study. These were both single-arm studies, and the observed outcomes may be affected by the prognosis of the enrolled patients.

A testament to the tolerability of the regimen was the median duration of therapy of approximately 8.2 months, which is almost twice the median duration of therapy with FOLFIRINOX (3). The upper range of therapy was approximately 23 months. While patients did experience bone marrow suppression, this is an expected consequence of the exposure time to chemotherapy. There were no treatment-related deaths.

In conclusion, while the GTX-CI regimen offers patients an option that is reasonably well tolerated and efficacious, the study did not meet its primary objective. This exact regimen will not be carried into future studies. However, the observation that a subset of patients with metastatic PDAC survived almost 4 years or longer, in a population with many poorly differentiated tumors, supports further investigation to optimize the regimen in hopes of increasing the number of patients with metastatic pancreatic cancer with long-term survival.

## Supplementary Material

Supplementary Table 1 Supplementary Table 1: Representativeness

Supplementary Table 2 Supplementary Table 2: Related Adverse Events

Supplementary Table 3 Supplementary Table 3: Patient Characteristics

Supplementary Figure 1 Supplementary Figure 1: Consort

Supplementary Figure 2 Supplementary Figure 2: OS and PFS
