# Supplementary material for: Gemcitabine, Docetaxel, Capecitabine, Cisplatin, Irinotecan as First-line Treatment for Metastatic Pancreatic Cancer
Source: Cancer Res Commun. 2023 Aug 28;3(8):1672–7. doi: 10.1158/2767-9764.CRC-23-0230 (PMC10461640; doi:10.1158/2767-9764.CRC-23-0230)
Supplement: Supplementary Table 1 — Representativeness [file crc-23-0230-s01.pdf]

Supplementary Table 1: Representativeness of Study Participants

Cancer type(s)/subtype(s)/stage(s)/condition	Pancreatic adenocarcinoma
Considerations related to:	
Sex	There is a slight male predominance with an incidence of 1.3:1 (males: females).
Age	Metastatic pancreatic ductal adenocarcinoma (mPDAC) is diagnosed at a median age of 70 years.
Race/Ethnicity	In the United States, based upon the 2018 SEER data submission, the incidence of Black Americans is higher compared to White Americans. The incidence is 16.7 per 100,000 for Black males compared to 14.8 per 100,000 for White males. The incidence is 14.6 per 100,000 for Black women compared to 11.5 per 100,000 for White women.
Overall representativeness of this study	The sex distribution in our study was reflective of the general population with a male: female incidence of 1.35. The median age was slightly lower at 64years. Our study did have a higher proportion of White Americans compare to Black Americans (8:1).
