# Supplementary material for: Gemcitabine, Docetaxel, Capecitabine, Cisplatin, Irinotecan as First-line Treatment for Metastatic Pancreatic Cancer
Source: Cancer Res Commun. 2023 Aug 28;3(8):1672–7. doi: 10.1158/2767-9764.CRC-23-0230 (PMC10461640; doi:10.1158/2767-9764.CRC-23-0230)
Supplement: Supplementary Table 2 — Related Adverse Events [file crc-23-0230-s02.pdf]

Supplementary Table 2. Related Adverse Events in >10% of the Phase 2 Dose Expansion

	Grade 1/2<br>N	Grade 3/4<br>N	Total N=30<br>N (%)
**Blood and lymphatic system disorders**			
Anemia	0	18	18 (60%)
**Gastrointestinal disorders**			
Anorexia	7	1	8 (27%)
Abdominal pain	4	0	4 (13%)
Diarrhea	20	0	20 (67%)
Dry mouth	4	0	4 (13%)
Flatulence	5	0	5 (17%)
Gastroesophageal reflux disease	4	0	4 (13%)
Mucositis oral	8	0	8 (27%)
Nausea	13	1	14 (47%)
Vomiting	11	2	13 (43%)
**General disorders and administration site conditions**			
Edema	7	0	7 (23%)
Fatigue	16	0	16 (53%)
Fever	6	0	6 (20%)
**Investigations**			
Alanine aminotransferase increased	0	4	4 (13%)
Creatinine, increased	8	0	8 (27%)
Lymphocyte count decreased	0	12	12 (40%)
Neutrophil count decreased	0	18	18 (60%)
Platelet count decreased	0	11	11 (37%)
Weight loss	2	1	3 (10%)
White blood count decreased	0	14	14 (47%)
**Metabolism and nutrition disorders**			
Dehydration	5	0	5 (17%)
Hypocalcemia	2	1	3 (10%)
Hypokalemia	4	3	7 (23%)
Hypomagnesemia	17	3	20 (67%)
Hypophosphatemia	1	4	5 (17%)
**Musculoskeletal and connective tissue disorders**			
Back pain	3	0	3 (10%)
Generalized muscle weakness	3	0	3 (10%)
**Nervous system disorders**			

	Grade 1/2<br>N	Grade 3/4<br>N	Total N=30<br>N (%)
Dizziness	5	0	5 (17%)
Dysgeusia	19	0	19 (63%)
Neuropathy	18	0	18 (60%)
**Renal and urinary disorders**			
Chronic kidney disease	1	2	3 (10%)
**Respiratory, thoracic and mediastinal disorders**			
Epistaxis	7	0	7 (23%)
Sore throat	3	0	3 (10%)
**Skin and subcutaneous tissue disorders**			
Alopecia	14	0	14 (47%)
Erythema	3	0	3 (10%)
Rash	8	0	8 (27%)
Skin hyperpigmentation	3	0	3 (10%)
