# Supplementary material for: Gemcitabine, Docetaxel, Capecitabine, Cisplatin, Irinotecan as First-line Treatment for Metastatic Pancreatic Cancer
Source: Cancer Res Commun. 2023 Aug 28;3(8):1672–7. doi: 10.1158/2767-9764.CRC-23-0230 (PMC10461640; doi:10.1158/2767-9764.CRC-23-0230)
Supplement: Supplementary Table 3 — Patient Characteristics [file crc-23-0230-s03.pdf]

Supplementary Table 3. Patient Characteristics

Phase	Dose Level	ID	On Study Age	Gender	Race	Pancreas Cancer Site	Differentiation	Liver Mets	Lung only	BRCA Mutation	KRAS Mutation	Baseline ECOG	Baseline Ascites	Baseline CA19-9	NLR	OS (months)	OS Censor	Best response	Surgical Resection
I	Dose level 1	P11	63	Female	White	Body/Tail	Poorly differentiated	Yes	No	UNK	UNK	1	No	11592	4.49	14.58726899	1	PD	No
I	Dose level 1	P23	65	Male	White	Head/Neck/Uncinate	Moderately differentiated	Yes	No	UNK	UNK	1	Yes	>400000.0	7.15	0.722792608	1	NE	No
I	Dose level 1	P36	67	Female	White	Head/Neck/Uncinate	Poorly differentiated	Yes	No	UNK	UNK	0	No	605.1	5.92	4.960985626	1	PR	No
I	Dose level 1	P38	72	Female	White	Head/Neck/Uncinate	Poorly differentiated	Yes	No	UNK	UNK	1	No	2.1	3.13	8.837782341	1	SD	No
I	Dose level 1	P39	73	Male	White	Body/Tail	Poorly differentiated	No	No	UNK	p.G12R	1	No	5133.5	2.60	25.79055441	1	SD	No
I	Dose level 1	P47	47	Female	White	Body/Tail	Poorly differentiated	Yes	No	UNK	UNK	1	Yes	2032.3	2.73	14.32443532	1	PR	No
2	Dose level 1a	P1	69	Male	White	Head/Neck/Uncinate	Moderately differentiated	Yes	No	No	p.G12R	0	No	255	3.09	19.97535934	1	PR	Yes
2	Dose level 1a	P10	65	Female	White	Head/Neck/Uncinate	Unknown	Yes	No	UNK	UNK	1	No	16439.5	2.56	5.848049281	1	PD	No
2	Dose level 1a	P12	67	Female	White	Body/Tail	Poorly differentiated	No	No	No	p.Q61R	0	No	41.8	2.79	21.65092402	1	SD	Yes
2	Dose level 1a	P13	68	Female	White	Head/Neck/Uncinate	Moderately differentiated	Yes	No	No	p.G12D	0	No	352.8	2.15	33.08418891	0	PR	Yes
2	Dose level 1a	P14	60	Male	White	Body/Tail	Unknown	Yes	No	UNK	p.G12V	0	Yes	152255.8	4.83	8.377823409	1	PR	No
2	Dose level 1a	P15	72	Male	White	Head/Neck/Uncinate	Moderately differentiated	Yes	No	No	UNK	1	No	32445.5	8.60	8.542094456	1	PR	No
2	Dose level 1a	P16	64	Male	White	Body/Tail	Poorly differentiated	Yes	No	No	UNK	1	Yes	13260.3	9.44	6.045174538	1	SD	No
2	Dose level 1a	P18	63	Female	White	Head/Neck/Uncinate	Poorly differentiated	Yes	No	UNK	UNK	1	No	1448.1	6.02	8.936344969	1	SD	No
2	Dose level 1a	P19	67	Female	Asian	Head/Neck/Uncinate	Poorly differentiated	Yes	No	No	UNK	1	No	<1	3.26	45.17453799	0	CR	No
2	Dose level 1a	P2	69	Male	White	Body/Tail	Poorly differentiated	Yes	No	UNK	UNK	1	No	9.8	1.43	2.95687885	1	PD	No
2	Dose level 1a	P20	67	Female	Black	Body/Tail	Moderately differentiated	Yes	No	No	p.G12D	1	No	99.3	6.89	7.293634497	1	SD	No
2	Dose level 1a	P21	45	Male	White	Body/Tail	Moderately differentiated	Yes	No	UNK	p.G12D	0	No	5882.1	3.83	11.17043121	1	PR	No
2	Dose level 1a	P22	68	Male	White	Body/Tail	Moderately differentiated	Yes	No	No	p.G12V	0	No	317.6	3.91	29.79876797	0	PR	Yes
2	Dose level 1a	P24	56	Female	White	Body/Tail	Poorly differentiated	No	No	UNK	UNK	1	No	268.7	1.23	8.31211499	1	PR	No
2	Dose level 1a	P25	68	Male	Black	Body/Tail	Poorly differentiated	Yes	No	UNK	UNK	1	Yes	<1	1.97	21.09240246	1	SD	No
2	Dose level 1a	P26	66	Male	White	Body/Tail	Poorly differentiated	Yes	No	BRCA2 (6174delT)	UNK	1	No	383.2	4.95	41.0349076	0	PR	Yes
2	Dose level 1a	P27	48	Male	White	Body/Tail	Moderately differentiated	Yes	No	UNK	UNK	0	No	18.9	5.46	5.650924025	1	PD	No
2	Dose level 1a	P28	58	Male	White	Body/Tail	Moderately differentiated	No	No	No	p.G12D	1	Yes	103.3	2.44	13.9301848	1	SD	No
2	Dose level 1a	P29	71	Male	White	Head/Neck/Uncinate	Poorly differentiated	Yes	No	UNK	UNK	1	No	<1	6.42	10.71047228	1	PR	No
2	Dose level 1a	P3	42	Male	White	Head/Neck/Uncinate	Moderately differentiated	Yes	No	UNK	UNK	0	No	78.4	4.49	5.848049281	1	PR	No
2	Dose level 1a	P30	54	Male	White	Head/Neck/Uncinate	Poorly differentiated	Yes	No	UNK	UNK	1	No	629.6	3.15	8.969199179	1	SD	No
2	Dose level 1a	P32	50	Female	White	Head/Neck/Uncinate	Moderately differentiated	Yes	No	No	UNK	0	No	177.9	1.17	17.34702259	1	PR	No
2	Dose level 1a	P33	48	Female	White	Head/Neck/Uncinate	Poorly differentiated	Yes	No	UNK	p.G12V	1	No	566.7	2.58	10.87474333	1	SD	No
2	Dose level 1a	P35	58	Male	White	Body/Tail	Moderately differentiated	Yes	No	UNK	UNK	1	Yes	99862.3	3.92	16	1	PR	No
2	Dose level 1a	P40	66	Male	White	Head/Neck/Uncinate	Poorly differentiated	Yes	No	UNK	UNK	1	No	24902.7	3.73	2.36550308	1	NE	No
2	Dose level 1a	P41	52	Male	White	Body/Tail	Poorly differentiated	No	No	No	p.G12R	1	Yes	203.5	3.58	36.13963039	0	PR	Yes
2	Dose level 1a	P43	74	Male	White	Head/Neck/Uncinate	Moderately differentiated	Yes	No	UNK	UNK	1	No	7805.3	3.33	40.14784394	0	PR	No
2	Dose level 1a	P44	50	Female	White	Head/Neck/Uncinate	Unknown	Yes	No	UNK	p.G12V	0	Yes	21,831.60	6.12	5.519507187	1	SD	No
2	Dose level 1a	P45	69	Male	Black	Body/Tail	Moderately differentiated	No	No	UNK	UNK	0	No	993.9	3.82	12.74743326	1	PR	No
2	Dose level 1a	P6	58	Female	White	Body/Tail	Poorly differentiated	Yes	No	No	UNK	1	No	7059.1	2.57	35.31827515	1	CR	No
I	Dose level 1b	P34	60	Male	White	Body/Tail	Poorly differentiated	Yes	No	No	UNK	1	No	29786.4	5.96	17.60985626	1	PR	No
I	Dose level 1b	P37	71	Male	White	Head/Neck/Uncinate	Moderately differentiated	Yes	No	UNK	UNK	1	Yes	39129.5	5.15	8.344969199	1	PR	No
I	Dose level 1b	P5	37	Female	White	Body/Tail	Poorly differentiated	Yes	No	No	p.G12D	1	Yes	2003.6	4.17	21.81519507	1	PR	No
I	Dose level 2	P17	63	Male	Black	Head/Neck/Uncinate	Poorly differentiated	Yes	No	UNK	UNK	0	No	97.2	2.27	21.84804928	1	NE	No
I	Dose level 2	P46	52	Male	Asian	Head/Neck/Uncinate	Moderately differentiated	Yes	No	UNK	UNK	1	No	3222.2	3.78	8.706365503	1	SD	No
I	Dose level 2	P7	55	Female	White	Head/Neck/Uncinate	Poorly differentiated	Yes	No	UNK	UNK	1	No	1652.9	4.38	5.519507187	1	PD	No
I	Dose level 2	P8	61	Male	White	Body/Tail	Poorly differentiated	Yes	No	UNK	UNK	0	No	925.3	2.84	5.9137577	1	SD	No
I	Dose Level 3	P31	64	Female	Black	Head/Neck/Uncinate	Poorly differentiated	Yes	No	UNK	UNK	1	No	8523	3.50	10.05338809	1	PR	No
I	Dose Level 3	P4	68	Female	White	Body/Tail	Unknown	Yes	No	UNK	UNK	1	No	1532.3	3.63	6.636550308	1	SD	No
I	Dose level 3	P42	53	Female	White	Body/Tail	Poorly differentiated	Yes	No	UNK	UNK	0	No	2.1	4.81	9.954825462	1	PR	No
I	Dose Level 3	P9	70	Male	White	Body/Tail	Moderately differentiated	Yes	No	BRCA1 (p.Glu143*)	UNK	1	No	8.1	4.03	21.12525667	1	PR	No
