# Supplementary figures and images for: Gemcitabine, Docetaxel, Capecitabine, Cisplatin, Irinotecan as First-line Treatment for Metastatic Pancreatic Cancer
Source: Cancer Res Commun. 2023 Aug 28;3(8):1672–7. doi: 10.1158/2767-9764.CRC-23-0230 (PMC10461640; doi:10.1158/2767-9764.CRC-23-0230)

## CONSORT 2010 Flow Diagram

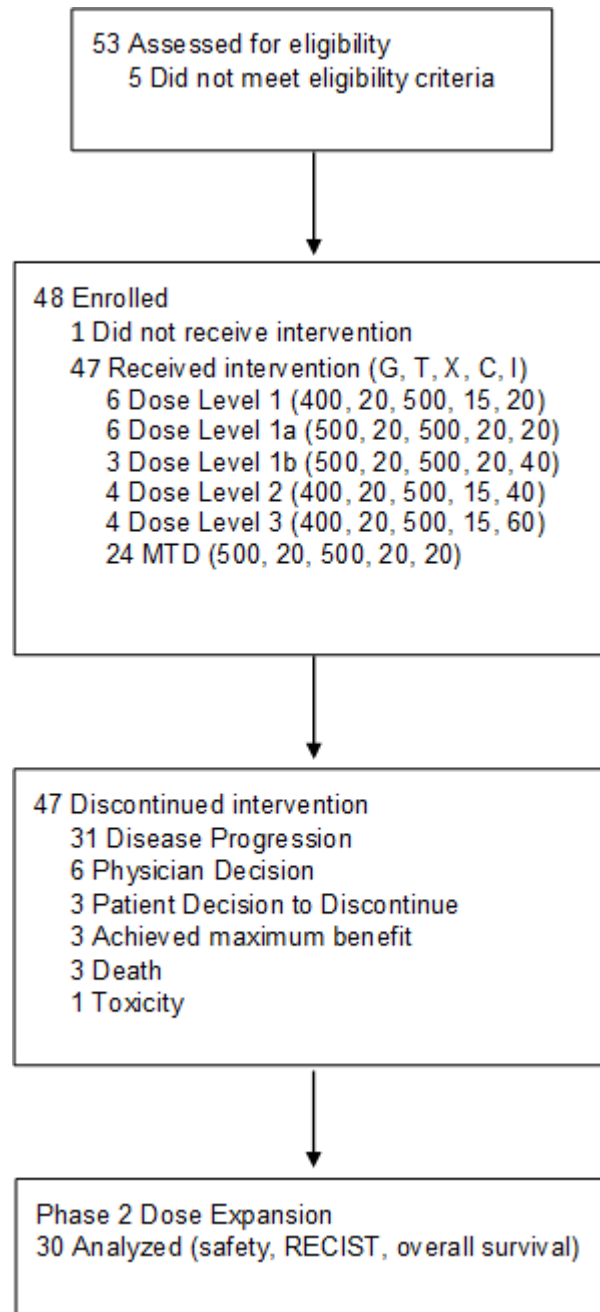

Supplementary Figure 1. CONSORT Diagram

Supplement: Supplementary Figure 1 — Consort [file crc-23-0230-s04.pdf]
