# Supplementary material for: Gemcitabine, Docetaxel, Capecitabine, Cisplatin, Irinotecan as First-line Treatment for Metastatic Pancreatic Cancer
Source: Cancer Res Commun. 2023 Aug 28;3(8):1672–7. doi: 10.1158/2767-9764.CRC-23-0230 (PMC10461640; doi:10.1158/2767-9764.CRC-23-0230)
Supplement: Supplementary Figure 2 — OS and PFS [file crc-23-0230-s05.pdf]

A. Overall Survival by Differentiation Status

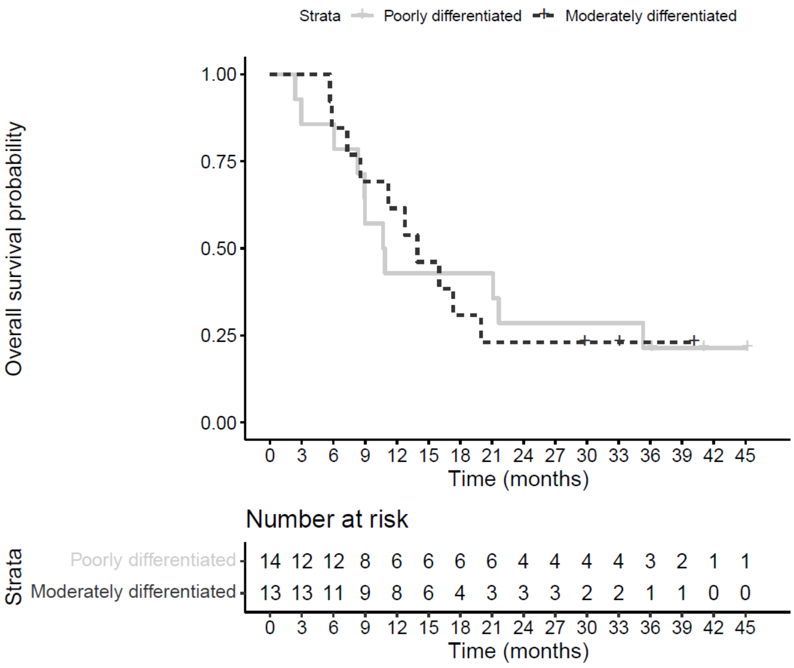

B. Progression-Free Survival by Differentiation Status

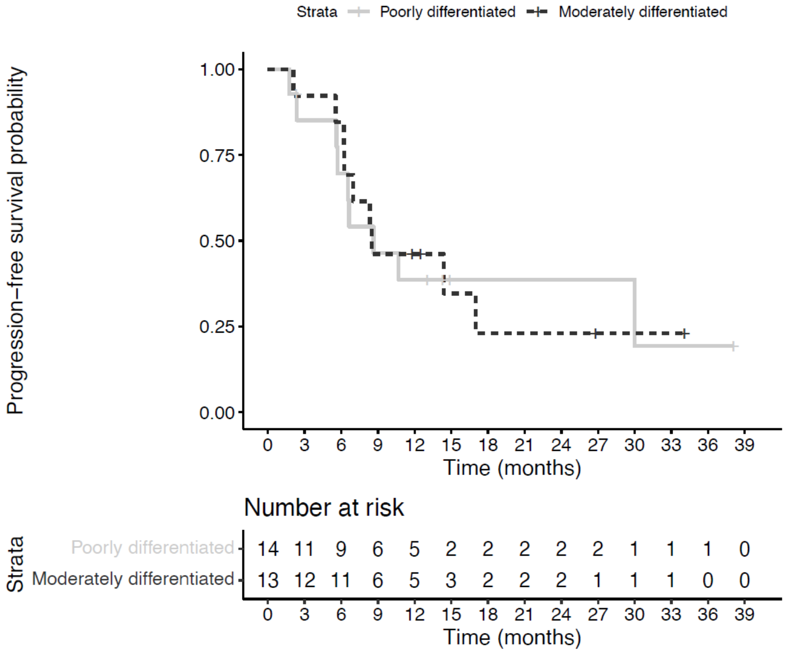

Supplementary Figure 2. Kaplan-Meier Estimates of Overall Survival and Progression-Free Survival in the Expansion Cohort by Differentiation Status. Panel A shows overall survival; the median was 13.9 and 10.8 months for well to moderately differentiated tumors and poorly differentiated tumors, respectively. Panel B shows progression-free survival; the median was 8.5 and 8.6 months for well to moderately differentiated tumors and poorly differentiated tumors, respectively.
